# Mechanistic Insights on Immune Activation-Driven Erectile Dysfunction: Evidence from Animal Models

**DOI:** 10.1007/s11930-026-00426-y

**Published:** 2026-08-26

**Authors:** Tiago T. Gonçalves, Raiana dos Anjos Moraes, Rinaldo Rodrigues dos Passos, Clinton Webb, Fernanda Priviero

**Affiliations:** 1 Cardiovascular Translational Research Center, Floyd School of Medicine, University of South Carolina, Columbia, SC 29208, USA; 2 Department of Biomedical Engineering, Molinaroli College of Engineering and Computing, University of South Carolina, Columbia, SC, USA; 3 Department of Cell Biology and Anatomy, Floyd School of Medicine, University of South Carolina, Columbia, SC, USA

**Keywords:** Erectile dysfunction, Inflammation, Inflammasomes, Cell death

## Abstract

**Purpose of Review:**

Erectile dysfunction (ED) has traditionally been attributed to impaired hemodynamics and reduced nitric oxide (NO) bioavailability. This review examines current evidence supporting immune activation and inflammation as shared mechanisms contributing to ED across diverse pathological conditions.

**Recent Findings:**

Experimental studies demonstrate that diabetes, hypertension, obesity, aging, nerve injury, and psychological stress are associated with activation of inflammatory pathways including NF-κB, Toll-like receptors, and the NLRP3 inflammasome. These pathways interact with oxidative stress and endothelial dysfunction to promote cytokine production, fibrosis, smooth muscle loss, and inflammatory cell death.

**Summary:**

Current evidence identifies inflammatory and innate immune pathways as convergent pathogenic drivers of ED across diverse etiologies. Rather than representing passive consequences of underlying disease, these mechanisms actively contribute to vascular, neural, and structural dysfunction, perpetuating processes that promote erectile impairment. Recognizing the central role of immune-mediated dysfunction in ED may facilitate the development of novel therapeutic strategies targeting disease mechanisms rather than symptoms alone.

## Introduction

Erectile dysfunction (ED) affects millions of men world-wide and is associated with aging and cardiometabolic diseases [1–5]. Earlier projections estimated that more than 320 million men worldwide would be affected by ED by 2025 [2]. Although this estimate has been widely cited, updated global epidemiological projections remain unavailable, highlighting the need for contemporary epidemiological assessments [6]. In addition, human studies also provide evidence that ED often precedes cardiovascular events, supporting the concept that ED may function as an early indicator of systemic vascular injury [3, 7–9]. This association is particularly evident in patients with diabetes, obesity, hypertension, and metabolic syndrome, conditions characterized by vascular dysfunction and low-grade chronic inflammation [3, 10, 11].

Historically, mechanistic explanations for ED focused mainly on alterations in penile blood flow, impairment of NO signaling, and smooth muscle dysfunction [12–14]. Penile erection depends on the integration of neural stimuli, reduced norepinephrine from adrenergic nerves, increased NO release by nitrergic nerves and the endothelium, activation of the soluble guanylate cyclase/cGMP pathway, and relaxation of cavernosal smooth muscle, events that allow increased blood inflow and functional venous occlusion [12–14]. Although alterations in these pathways are well established in ED, growing evidence indicates that chronic inflammation and innate immune activation contribute to the endothelial, neural, and smooth muscle dysfunction underlying these abnormalities. In fact, clinical and mechanistic studies have linked ED to elevated inflammatory mediators and endothelial dysfunction markers, including TNF-α, IL-6, C-reactive protein, adhesion molecules, and broader cytokine profiles [10, 11, 15–17]. The origin of this inflammatory state remains unclear, but its presence across diverse etiologies suggests a common upstream mechanism [17–19]. The danger model provides a framework in which cellular stress and tissue injury trigger innate immune activation, leading to sustained inflammation and tissue dysfunction [20]. Indeed, experimental evidence supports the concept that immune activation contributes to endothelial dysfunction, altered vascular tone, cavernosal fibrosis, smooth muscle loss, and neurovascular impairment [1, 11, 21, 22]. Therefore the sexual organs are particularly vulnerable to inflammation-mediated dysfunction because erectile physiology depends on precise coordination among endothelial, neuronal, and smooth muscle systems [12–14].

Animal models have been fundamental to this paradigm shift by enabling controlled disease induction, longitudinal tissue assessment, and genetic manipulation. These approaches have identified convergent inflammatory pathways across diverse disease models despite distinct initiating triggers [1, 11, 22]. This consistency suggests that ED involves conserved tissue responses rather than pathways specific to each disease.

This review focuses on the broader biological narrative emerging from experimental evidence: different systemic stressors converge toward a shared inflammatory phenotype, characterized by oxidative stress, innate immune activation, cytokine amplification, and progressive tissue remodeling [1, 11, 22, 23]. Understanding this convergence may enable the development of disease-modifying therapies that target the underlying biology of ED, rather than merely restoring erectile performance.

## Inflammatory Remodeling of Erectile Tissue

A central question arises from experimental models: why do different pathological states originating from distinct mechanisms produce similar erectile phenotypes [1, 11, 24–26]? The answer seems to reside in the conserved tissue responses they provoke. Animal models of ED show that different systemic conditions often converge on a common pattern of alterations in the corpus cavernosum, including oxidative stress, reduced NO bioavailability, endothelial dysfunction, inflammatory activation, apoptosis, fibrosis, and impaired cavernosal relaxation [1, 11, 24, 25, 27]. This section examines how oxidative stress, innate immune activation, and inflammasome signaling drive genital vascular and cavernosal remodeling across etiologies [11, 26–29].

The major pathways discussed throughout this review are summarized in Fig. 1, illustrating how diverse pathological conditions converge on common mechanisms involving oxidative stress, innate immune activation, inflammasome signaling, tissue remodeling, and erectile dysfunction.

## From Oxidative Stress to Innate Immune Activation

Different etiologies of ED appear to result in common alterations in the genital vasculature and cavernosal tissue, compromising NO bioavailability, cavernosal smooth muscle function, and favoring the development of fibrosis [1, 15, 30–35]. In different experimental models, such alterations are associated with reduced NOS activity and/or expression, post-translational modifications of endothelial nitric oxide synthase (eNOS), such as O-GlcNAcylation, and the participation of inflammatory mediators such as TNF-α, reinforcing the mechanistic overlap among distinct etiologies of ED [30–35].

Oxidative stress has emerged as a central mechanism linking diverse pathological conditions to ED [1, 30, 31]. Although transient increases in ROS can be tolerated in many vascular beds, sustained oxidative stress leads to endothelial dysfunction through chronic inflammatory remodeling triggered by persistent activation of innate immune pathways, favoring a self-perpetuating cycle of oxidative damage, endothelial activation, cytokine production, and tissue remodeling [1, 11, 15]. Diabetic, hypertensive, aged, and stress-exposed animals show increased cavernosal ROS production, reduced antioxidant defenses, and impaired NO bioavailability [30, 32]. Excessive oxidative stress contributes to amplification of the inflammatory environment by activating transcription factors (such as NF-κB), and by promoting mitochondrial dysfunction, endothelial injury, and cellular death or senescence programs [1, 11, 15, 25].

The genital vasculature and corpus cavernosum may be particularly vulnerable to these alterations because normal erectile physiology depends on tightly coordinated physiological processes [1, 11–13]. Even small dysfunctions in nitrergic signaling, endothelial integrity, or cavernosal smooth muscle relaxation produce proportionally amplified functional consequences [12, 13]. Among the pathways linking oxidative stress to inflammatory activation, innate immune receptors have received increasing attention.

Toll-like receptors (TLRs) have emerged as important early regulators of inflammatory signaling in erectile tissue. The TLR1/2 heterodimer promotes cavernosal hypercontractility and impairs relaxation through activation of RhoA/ Rho-kinase pathway, demonstrating that innate immune signaling can directly alter penile vascular tone independently of endothelial NO [36]. However, direct evidence of chronic remodeling driven by TLR2 in erectile tissue is still limited. In arteries, TLR4 activation promotes endothelial dysfunction and fibrosis through the MyD88/NF-κB pathway [37]. In hypertension-associated ED, angiotensin II and TLR4 signaling appear to act synergistically, reducing NO bioavailability and amplifying cavernosal dysfunction, suggesting that cardiovascular drugs targeting the renin-angiotensin system may exert benefits beyond blood pressure reduction, through suppression of innate immune activation [38].

In addition to classic cardiovascular risk factors, chronic ethanol exposure also induces cavernosal dysfunction through NADPH oxidase-derived ROS production, NF-κB activation, inflammation, and altered cavernosal smooth muscle reactivity [39]. These findings reinforce the concept that different metabolic, hemodynamic, and toxicological stressors trigger similar pathways of inflammatory remodeling. Therapeutically, this suggests that interventions targeting shared inflammatory mechanisms could benefit subgroups of patients regardless of the predominant etiology of ED.

In obesity-driven ED, mitochondrial DNA (mtDNA) released by dysfunctional adipose tissue activates macrophage-specific TLR9 signaling, directly impairing corpus cavernosum relaxation in mice fed a high-fat diet [40]. More recently, environmental exposures, such as polyethylene terephthalate microplastics, have been associated with ED through mitochondrial damage, mtDNA release, macrophage-mediated cGAS-STING activation, ferroptosis, and inflammation in the corpus cavernosum [41]. These findings indicate that beyond the traditional vascular risk factors, metabolic and environmental inflammation can directly impact ED.

This upstream activation of innate immune receptors sets the stage for downstream inflammatory amplification [36–42]. Once TLRs recognize their ligands and activate transcription factors, the inflammatory cascade becomes self-sustaining through the production of pro-inflammatory cytokines and the activation of additional inflammatory platforms [11, 15, 25], including the NLRP3 inflammasome [42].

## Inflammasome Signaling and Cytokine Amplification

Downstream of innate immune pathways, the NLRP3 inflammasome has emerged as an important integrative signaling platform, connecting cellular stress to inflammatory tissue injury [42, 43]. NLRP3 responds to multiple stress signals and converts them into coordinated inflammatory responses, including mitochondrial dysfunction, ROS accumulation, ATP release, lipid overload, potassium efflux, lysosomal damage, and intracellular damage-associated molecular patterns (DAMPs) [42, 43], explaining why inflammasome activation appears repeatedly in different models of ED, despite major differences in their initiating triggers [27–29, 44–46].

Inflammasome activation triggers pyroptosis, a lytic and pro-inflammatory form of cell death mediated by inflammatory caspases and pore formation by members of the gas-dermin family, especially gasdermin D. Pyroptosis involves membrane rupture and release of inflammatory mediators, such as IL-1β, IL-18, and immunogenic intracellular content, amplifying the local inflammatory response [47–49]. Experimental models demonstrate increased expression of NLRP3, ASC, caspase-1, IL-1β, IL-18, and/or markers of pyroptosis in erectile tissue exposed to chronic inflammatory stress [29, 44, 46]. In models associated with diabetes and obesity, pharmacological or molecular inhibition of NLRP3 reduces pyroptosis, preserves eNOS expression, improves the structural integrity of the corpus cavernosum, and favors recovery of erectile responses, linking inflammasome activation to disease progression [29, 45, 46]. Consistent with this concept, NLRP3 inhibition in diabetic ED also attenuates NF-κB signaling and oxidative stress while improving endothelial markers, further linking inflammasome activation to corporal endothelial dysfunction [50]. The therapeutic efficacy of inflammasome inhibition reinforces the idea that this pathway acts as an active component of the pathophysiology, and not as a passive biomarker of ED.

In diabetes-induced ED, neutrophil extracellular traps (NETs) amplify NLRP3-mediated pyroptosis, creating a positive inflammatory feedback loop in which immune activation promotes cell death and dying cells further intensify immune responses [44]. Similarly, in hypertensive models, upregulation of fatty acid synthase drives endothelial pyroptosis through NLRP3 activation and modulation of the Nrf2/HO-1 pathway, reinforcing pyroptosis as an active mechanism of propagation [28].

Beyond pyroptosis, growing evidence suggests that ferroptosis, cell death driven by iron-dependent lipid peroxidation, may also participate in cavernosal remodeling [51–53]. In models of diabetes, modulation of GPX4 and Nrf2/HO-1/GPX4 pathway reduce lipid peroxidation, oxidative stress, ferroptosis, and ED, suggesting that multiple forms of regulated cell death may simultaneously contribute to chronic inflammatory remodeling [51, 52]. Nevertheless, the temporal relationships and relative contributions of pyroptosis, ferroptosis, and apoptosis during ED progression remain incompletely understood [53].

However, the role of NLRP3 signaling may be more complex than initially assumed. NLRP3 can exert both contractile and relaxing effects in the mouse corpus cavernosum, depending on experimental context, level of activation, and redox state of the tissue, highlighting that signaling pathways that drive pathology when chronically activated often maintain physiological functions under basal conditions [26]. Consequently, the therapeutic challenge lies in selectively targeting its pathological activation while preserving its physiological roles.

Once activated, inflammasomes amplify inflammatory signaling through cytokine production. IL-1β and IL-18 recruit additional immune cells and directly compromise endothelial and smooth muscle function [42, 43, 49]. IL-18, in particular, has been implicated in suppressing NO/cGMP signaling even in the presence of PDE5 inhibitors in a model of high-fat diet-associated ED, potentially contributing to inadequate responses to first-line therapy [29]. TNF-α, generated through both inflammasome-dependent and inflammasome-independent pathways, further amplifies vascular dysfunction by reducing eNOS and nNOS expression, promoting oxidative stress, and activating RhoA/ROCK signaling, a key vasoconstrictor pathway limiting erection [15, 54].

Therefore, this cytokine-rich inflammatory environment creates favorable conditions for progressive tissue remodeling, including apoptosis, fibrosis, alteration in the smooth muscle/collagen ratio, and loss of cavernosal relaxation capacity [29, 44].

## Fibrosis, Senescence, and Irreversible Tissue Remodeling

While transient oxidative stress and cytokine signaling in erectile tissue can recover cavernosal function after resolution of the initial trigger [26, 52], persistent inflammatory activation promotes structural alterations that become self-sustaining and progressively less reversible over time [1, 55–57]. These alterations include cavernosal fibrosis, smooth muscle apoptosis, endothelial dysfunction, alteration of the smooth muscle/collagen ratio, and cellular senescence, processes that compromise acute erectile responses and progressively reduce the tissue capacity to recover its normal structure and function [1, 55–58].

Fibrosis represents one of the most clinically relevant forms of inflammatory remodeling. Transforming growth factor-β (TGF-β) signaling, which is frequently activated in response to oxidative stress, inflammation, and chronic tissue injury, drives excessive extracellular matrix deposition, myofibroblast differentiation, and collagen accumulation [55, 56, 59]. In models of cavernous nerve injury, mitochondrial dysfunction, oxidative stress, and apoptosis of corpus cavernosum smooth muscle cells contribute to smooth muscle loss and corporal fibrosis [60, 61]. This fibrotic remodeling may limit the efficacy of standard therapies. Even when NO/cGMP signaling is pharmacologically amplified by PDE5 inhibitors, tissue with advanced fibrosis fails to relax adequately, since its structural properties have been altered by collagen deposition, smooth muscle loss, and morpho-structural disorganization of the corpus cavernosum [55–57, 62].

In addition to fibrosis, defective tissue repair actively contributes to ED progression. Bone morphogenetic protein 2 (BMP2), a growth factor implicated in angiogenesis, tissue repair, and neurovascular regeneration, partially improves structural and neurovascular parameters in diabetes, but may not completely restore erectile function in a chronically altered metabolic environment [63]. This regenerative failure suggests that ED progression involves destructive inflammatory remodeling and impaired reparative responses, particularly in metabolic states in which chronic inflammation and oxidative stress limit the capacity for tissue regeneration [1, 63, 64].

Cellular senescence, a state of stable growth arrest accompanied by cellular remodeling and pro-inflammatory secretory phenotype, further contributes to tissue deterioration [65, 66]. In experimental neurogenic/diabetic ED, IL-17 A induces senescence of corpus cavernosum smooth muscle cells through activation of the mTORC2-ACACA pathway, connecting inflammatory cytokine signaling to cellular aging programs and cavernosal fibrosis [19]. Senescent cells can perpetuate inflammation by secreting cytokines, chemokines, growth factors, and matrix metalloproteinases collectively termed the senescence-associated secretory phenotype (SASP) [65, 66]. This creates a vicious cycle: inflammation induces senescence, and senescent cells, in turn, maintain a pro-inflammatory environment, establishing a feedforward circuit that may persist even after attenuation of the original inflammatory trigger [19, 65, 66].

Furthermore, different etiologies appear to culminate in similar fibrotic, apoptotic, and degenerative outcomes. Radiation-induced ED, a relevant complication after pelvic radiotherapy for prostate and colorectal cancers, demonstrates oxidative stress, apoptosis, fibrosis, endothelial impairment, and impaired neurovascular integrity in experimental models [67] Similarly, in diabetes, HIF-1α-mediated hypoxic signaling and activation of the RhoA/ROCK pathway promote contractile dysfunction and structural degeneration of the corpus cavernosum [68–70]. This similarity among the pathways across different triggers reinforces the concept that the pathophysiology of ED reflects a limited repertoire of tissue responses to different chronic stressors, rather than mechanisms completely specific to each disease [1, 55, 58].

It is important to emphasize that, once fibrosis, senescence, and smooth muscle loss progress sufficiently, erectile tissue loses a substantial part of its capacity to respond to therapies that would be effective at earlier stages of ED. This progressive loss of reversibility highlights the importance of early interventions targeting initial inflammatory mechanisms before structural remodeling becomes established, a therapeutic window that probably narrows as the disease progresses [1, 55–57, 62].

## Disease-Specific Triggers of Immune Activation

Common inflammatory mechanisms are observed across multiple forms of ED, however, the underlying disease context remains important because different systemic conditions activate these pathways through distinct biological factors [1, 11, 24, 25, 29]. Understanding these disease-specific entry points may be clinically important for identifying patients at higher risk, recognizing phenotypes more likely to progress, and developing targeted early interventions [1, 11, 24, 25, 64].

### Immunometabolic Erectile Dysfunction

Diabetes remains one of the most studied conditions in the context of inflammatory ED and is associated with a substantially higher prevalence of ED compared with men without diabetes [64, 71]. Hyperglycemia induces oxidative stress through multiple mechanisms, including glucose autoxidation, formation of advanced glycation end-products, increased flux through the polyol pathway, activation of the hexosamine pathway, and mitochondrial superoxide overproduction [72–75]. Beyond these metabolic disturbances, chronic hyperglycemia favors immunometabolic remodeling characterized by endothelial dysfunction, reduced NO bioavailability, inflammatory cell infiltration, cytokine production, apoptosis, alterations of cavernosal smooth muscle, and progressive tissue fibrosis [18, 32, 64, 71, 76] Metabolic disturbances can also promote O-GlcNAcylation of eNOS, a post-translational modification intensified by hyperglycemia, impairing eNOS activation even when upstream signaling pathways remain intact [32]. This modification exemplifies how metabolic stress can translate into sustained enzymatic dysfunction, independently of acute substrate availability. Oxidative stress also induces NETs, web-like structures composed of extracellular DNA associated with antimicrobial proteins, which have been recognized as amplifiers of diabetic vascular injury [44, 77]. In diabetic rats, NET formation correlates with NLRP3 inflammasome activation and pyroptosis in the corpus cavernosum [44]. Degradation of NETs with DNase reduces inflammatory injury and partially preserves erectile function, positioning innate immune cell activation as a direct pathogenic mechanism, and not merely as a secondary consequence of hyperglycemia [44]. Recent multiomics evidence further supports the convergence of inflammatory and structural mechanisms in diabetic ED. Comparative analysis of type 1 and type 2 diabetic models identified shared transcriptional alterations involving extracellular matrix-receptor interactions, focal adhesion, and PI3K-Akt signaling, together with increased macrophage infiltration and apoptosis, suggesting that inflammation and extracellular matrix remodeling represent conserved features across diabetic ED phenotypes [78]. Although frequently coexisting with diabetes, obesity itself contributes to inflammatory stress through dysfunctional adipose tissue. Adipocyte hypertrophy, tissue hypoxia, macrophage infiltration and polarization, as well as alterations in fatty acid metabolism, sustain a state of low-grade systemic inflammation characterized by increased levels of TNF-α, IL-6, MCP-1, and free fatty acids [79–81]. As discussed previously, mitochondrial DNA released by dysfunctional adipose tissue activates TLR9 in macrophages, directly impairing corpus cavernosum relaxation in mice fed a high-fat diet [40]. Therapeutic interventions targeting oxidative stress, such as administration of nitro-oleic acid, reduce ROS generation, activate the NO/cGMP pathway, reduce apoptosis, and improve erectile function in diabetic models, supporting the concept that metabolic inflammation represents a modifiable therapeutic target [68, 82].

Metabolic syndrome, characterized by the clustering of obesity, hypertension, dyslipidemia, and insulin resistance, may represent a particularly marked form of immunometabolic dysfunction [83, 84]. Nonalcoholic steatohepatitis (NASH), the hepatic manifestation of metabolic syndrome, is associated with systemic inflammation, increased circulating TNF-α levels, and impaired cavernosal relaxation in experimental models [82]. Evidence suggests that this systemic inflammatory environment, rather than hepatic dysfunction in isolation, appears to mediate erectile impairment, since TNF-α neutralization restores erectile responses independently of hepatic histology [82].

### Vascular and Hemodynamic Stress

Hypertension and atherosclerosis, traditionally viewed as hemodynamic disorders, are increasingly recognized as conditions associated with chronic vascular inflammation, oxidative stress, endothelial dysfunction, and tissue remodeling [30, 38, 85, 86]. In atherosclerotic models of ED, chronic ischemia generates persistent oxidative stress, cavernosal smooth muscle dysfunction, and fibrosis [30]. Antioxidant interventions reduce cavernosal fibrosis and preserve erectile function, demonstrating that oxidative injury actively participates in structural remodeling, rather than simply reflecting reduced blood flow [30, 85].

In hypertensive models, angiotensin II and TLR4 signaling act synergistically to amplify inflammatory dysfunction and reduce NO bioavailability in the corpus cavernosum [38]. This interaction has clinical relevance, suggesting that inhibitors of the renin-angiotensin system may exert benefits beyond blood pressure reduction, through suppression of vascular inflammation and oxidative stress [38, 86]. In addition, fatty acid synthase-driven endothelial pyroptosis in hypertension positions metabolic-inflammatory crosstalk as a relevant component even in classically “hemodynamic” forms of ED [28]. More recently, an endothelin-1 (ET-1)/membrane depolarization/NLRP3 inflammasome axis has been identified in hypertension-associated ED. In DOCA/salt hypertensive mice, pharmacological activation of endothelial KCa2.2/2.3 channels reduced NLRP3 activation and IL-1β signaling while improving cavernosal relaxation and erectile function, identifying endothelial ion-channel dysfunction as an additional link between hypertension and innate immune activation in erectile tissue [87].

Galectin-3, a β-galactoside-binding lectin implicated in cardiovascular inflammation and fibrosis, also promotes cavernosal fibrosis, endothelial dysfunction, and inflammation through activation of the TLR4/MyD88/NF-κB pathway in atherosclerotic models [37]. Galectin-3 inhibition reduces inflammation and fibrosis and improves erectile function, suggesting that shared fibrotic and inflammatory mechanisms may operate in both cardiovascular and erectile tissues [37].

### Aging, Senescence, and Neurogenic Injury

Aging is associated with a state of low-grade chronic inflammation termed “inflammaging,” characterized by increased levels of inflammatory mediators even in the absence of infection or manifest disease [88, 89]. This chronic inflammatory state involves innate and adaptive immune activation, as well as the participation of endothelial cells, fibroblasts, adipocytes, and senescent cells, and may contribute to vascular dysfunction and progressive tissue deterioration [88,89]. Aged rats demonstrate increased levels of TNF-α, CRP, MCP-1, and ICAM-1 in serum and/or corpus cavernosum, together with reduced eNOS and nNOS expression [33]. Neutralization of TNF-α partially restores NOS expression and improves cavernosal reactivity in aged animals, suggesting direct participation of cytokine-mediated inflammation in aging-related erectile decline [15, 33].

Similarly, cavernous nerve injury-induced ED, which is frequently associated with radical prostatectomy for prostate cancer, is triggered by neurovascular dysfunction followed by hypoxia, oxidative stress, apoptosis, fibrosis, and maladaptive remodeling of the corpus cavernosum [27, 63]. On the other hand, activation of the Nrf2 pathway through dimethyl fumarate reduces oxidative stress, suppresses NLRP3-mediated pyroptosis, and partially preserves erectile function after bilateral cavernous nerve injury [27]. Additionally, therapeutic interventions targeting mitochondrial dysfunction, cavernosal smooth muscle apoptosis, and corporal fibrosis improve structural and functional parameters after nerve injury, although complete functional recovery remains difficult to achieve in advanced models [60, 61, 90]. Interestingly, deficient regeneration may be as important as active degeneration in the progression of ED. Although BMP2 promotes neurovascular regeneration and reduces fibrosis in diabetic models, it does not completely restore erectile function in a chronically altered metabolic environment [63]. This suggests that chronic metabolic inflammation may create a tissue environment partially refractory to regenerative signals, especially when diabetes, nerve injury, fibrosis, and endothelial dysfunction coexist [61, 63].

### Atherosclerotic Vascular Injury

Atherosclerosis is a major vascular cause of ED because it narrows and stiffens the arteries that supply blood to the penis [91]. Normal erectile function depends on adequate arterial inflow and relaxation of penile smooth muscle mediated by nitric oxide (NO). As atherosclerotic plaques develop within the penile and pelvic arteries, blood flow becomes restricted, reducing the ability to achieve and maintain an erection [92]. Because penile arteries are relatively small in diameter, they may become symptomatic before larger coronary or carotid arteries, making ED an important early marker of systemic cardiovascular disease [7].

Inflammation plays a central role in both atherosclerosis and the development of ED [93, 94]. Endothelial injury caused by risk factors such as hypertension, diabetes, smoking, and hyperlipidemia triggers an inflammatory response characterized by the recruitment of monocytes, macrophages, and T lymphocytes into the vessel wall [95, 96]. These cells release pro-inflammatory cytokines and reactive oxygen species that promote plaque formation and impair endothelial function. The resulting oxidative stress decreases NO bioavailability, limiting vasodilation and reducing penile blood flow. Chronic vascular inflammation also contributes to plaque progression and arterial stiffness, further compromising erectile function. Thus, ED is increasingly recognized as a vascular disorder and inflammatory condition linked to the systemic processes underlying atherosclerosis [97].

### Psychological Stress and Neuroimmune Signaling

Psychological stress was historically categorized separately from organic ED, implying distinct underlying mechanisms. Emerging evidence challenges this dichotomy. Chronic mild unpredictable stress in rodents produces cavernosal dysfunction accompanied by inflammatory cytokines and chemokines, reduced penile eNOS and nNOS expression and impaired corpus cavernosum reactivity [34, 35, 98]. Neutralization of TNF-α prevents or attenuates stress-induced ED, demonstrating that psychological stress can produce organic inflammatory alterations rather than merely behavioral dysfunction [34, 35].

The mechanism connecting psychological stress to cavernosal inflammation likely involves neuroendocrine-immune interaction. Chronic stress activates the hypothalamic-pituitary-adrenal axis and the sympathetic nervous system, modulating systemic inflammation and vascular function [99, 100]. Sustained exposure to glucocorticoids may favor glucocorticoid resistance, while prolonged sympathetic activation may stimulate cytokine production by immune cells and alter peripheral neurovascular regulation [101–103].

Social stress models reveal impaired parasympathetic neurotransmission and nitrergic signaling, suggesting that neurovascular dysfunction may develop independently of overt systemic inflammatory activation [104]. This suggests that stress-induced ED involves both central nervous system alterations and peripheral functional remodeling. However, as highlighted in the Basic Science and Translational Research recommendations from the International Consultation on Sexual Medicine (ICSM), the immune response in psychosocial disorder-associated ED has not yet been investigated [105]. Therapeutically, the NO-cGMP pathway appears to remain a relevant target in stress-induced ED, as tadalafil improves parameters of sexual behavior and sexual function in rats subjected to chronic mild unpredictable stress, suggesting that amplification of the cGMP pathway can partially overcome stress-induced functional impairment [106].

Similarly, chronic sleep deprivation - another form of physiological stress increasingly recognized in modern society - induces ED in rats, accompanied by increased oxidative stress, tissue apoptosis, endothelial dysfunction, and cavernosal fibrosis [107]. The similarity among the phenotypes of ED induced by psychological stress, sleep deprivation, and metabolic alterations reinforces a unifying concept: different chronic stressors can activate similar pathways of inflammatory, oxidative, and neurovascular remodeling in erectile tissue [34, 35, 107].

## Therapeutic Implications and Translational Challenges

The evidence discussed throughout this review supports the concept that ED is not merely an isolated vascular or neurological disorder, but rather a manifestation of chronic inflammation and tissue remodeling. This pathophysiological framework has important therapeutic implications while also highlighting significant translational challenges.

PDE5 inhibitors remain the first-line therapy for ED and demonstrate efficacy in multiple pathological contexts [108–110]. These agents act by amplifying cGMP signaling to promote smooth muscle relaxation, increasing NO-dependent cGMP accumulation and favoring caver-nosal relaxation [108, 111] However, PDE5 inhibitors do not directly modify the initial inflammatory mechanisms. Patients with severe endothelial dysfunction, extensive fibrosis, or marked smooth muscle loss may respond inadequately because the structural substrate necessary for cGMP-mediated relaxation is compromised [55, 112].

Beyond the classic pro-erectile effect, PDE5 inhibitors may exert additional anti-inflammatory effects. Sildenafil reduces activation of the NLRP3 inflammasome, caspase-1, gasdermin D, and IL-18 production in high-fat diet-associated ED [29]. Tadalafil improves parameters of sexual function in chronic stress models, suggesting that amplification of the cGMP pathway partially compensates for stress-induced alterations [106]. In addition, tadalafil exerts systemic pleiotropic effects in heart failure, improving cardiac, renal, and lower urinary tract dysfunctions, probably through mechanisms involving vascular modulation and tissue protection [113]. These observations suggest that PDE5 inhibitors function as multifactorial vascular modulators rather than simple vasodilators, although most mechanistic evidence derives from animal studies [29, 106, 108, 113].

More targeted anti-inflammatory strategies have demonstrated promising results in experimental models but have not yet been translated into clinical practice for ED [1, 11, 27, 63]. Nrf2 activators, such as dimethyl fumarate, reduce oxidative stress, suppress NLRP3-mediated pyroptosis, and partially preserve erectile function after bilateral cavernous nerve injury [27]. Similarly, interventions targeting ferroptosis through modulation of the Nrf2/HO-1/GPX4 pathway improve erectile responses in diabetes [51, 52]. Natural products with antioxidant and vasoprotective properties, including medicinal plants traditionally used for male sexual dysfunction such as *Pfaffia glomerata*, *Anemopaegma arvense*, *Trichilia catigua*, and *Ptychopetalum olacoides*, have demonstrated beneficial effects in preclinical models of ED [114]. Proposed mechanisms include increased NO bioavailability, PDE5 inhibition, antioxidant activity, and modulation of neuroendocrine pathways. However, rigorous clinical evidence supporting their efficacy and safety remains limited.

Direct inflammasome inhibition represents an attractive but challenging therapeutic target. Although NLRP3 inhibitors improve inflammatory parameters in several experimental models and are currently under clinical development for inflammatory, metabolic, and neurodegenerative diseases, their specific application in ED has not yet been systematically evaluated in humans [115, 116] In addition, concerns related to selectivity, off-target effects, long-term safety, and possible interference with physiological immune responses remain relevant in the development of these drugs [115, 117]. More selective approaches targeting specific downstream effectors, such as IL-1β or IL-18 neutralization, may offer better therapeutic indices in some contexts, but their role in ED remains largely unexplored.

TNF-α neutralization, already approved for the treatment of inflammatory diseases such as rheumatoid arthritis, improves erectile function in experimental models of aging and stress, reinforcing the role of this mediator as a link between inflammation, reduced NOS, and cavernosal dysfunction [33, 98]. This therapeutic approach directly targets a proximal inflammatory mediator, and not only downstream pathways. However, concerns related to infection risk, cost, and long-term safety have limited its broader adoption for ED, despite its mechanistic rationale [15, 33].

Several factors hinder the translation of experimental findings into clinical practice [1, 11, 24, 29]. Most studies use young, genetically homogeneous animals subjected to diseases induced over defined periods, whereas human ED develops over years, in a context of multiple comorbidities, medication use, vascular aging, and hormonal and metabolic heterogeneity [1, 11, 24]. In addition, rodent models frequently assess erectile responses under anesthesia and electrical stimulation of the cavernous nerve, highly controlled conditions that do not fully reflect the psychological, hormonal, vascular, inflammatory, and relational dimensions of human sexual activity [24].

The ideal timing of intervention and patient selection also remain uncertain. Individuals in early stages may benefit more from disease-modifying approaches, whereas cases with advanced fibrosis will probably require combined strategies aimed at both inflammation and structural restoration [55, 112]. Furthermore, because ED involves simultaneous activation of multiple inflammatory networks, blockade of isolated mediators may be insufficient in the face of persistent compensatory pathways [1, 11].

Despite these limitations, this inflammatory interpretation of ED offers an important conceptual contribution: it repositions ED as a possible early manifestation of systemic vascular, immunometabolic, and inflammatory dysfunction, and not merely as an isolated urological complaint [1, 3, 11]. This perspective broadens its clinical implications, suggesting that men with erectile symptoms may benefit from systematic cardiovascular and metabolic assessment, in addition to early management of inflammatory factors and vascular risk, even in the absence of evident traditional risk factors [3, 7, 8].

## Conclusion and Future Perspectives

In conclusion, available evidence suggests that ED may represent an early manifestation of systemic vascular, immuno-metabolic, and inflammatory dysfunction. Despite diverse initiating factors, including metabolic disease, aging, neurogenic injury, and psychological stress, experimental models consistently demonstrate convergence on common inflammatory and tissue remodeling pathways. Although important questions remain regarding the temporal sequence of these events and their validation in human disease, pathways such as TLRs, NLRP3 inflammasome signaling, regulated cell death, and cellular senescence represent promising therapeutic targets. Future studies should identify biomarkers of inflammatory burden and determine whether targeting these mechanisms can modify disease progression rather than simply improve erectile performance. Beyond sexual dysfunction, this framework positions ED as a potential early indicator of systemic disease and an opportunity for preventive intervention.

## Figures and Tables

**Fig. 1:**
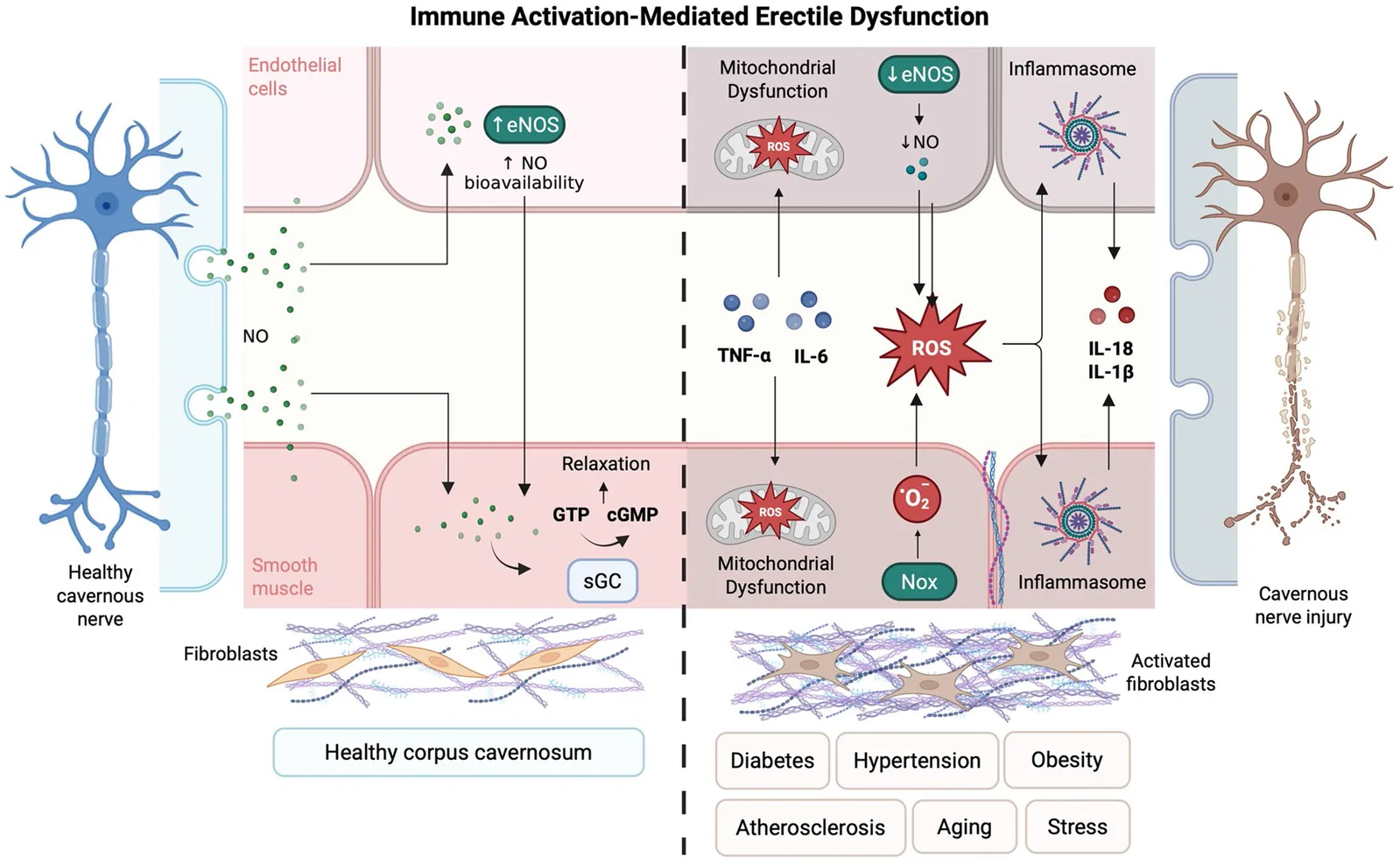
Proposed mechanisms underlying immune activation-mediated erectile dysfunction. Diverse pathological conditions, including diabetes, hypertension, obesity, aging, atherosclerosis, psychological stress, and cavernous nerve injury, converge on common pathways involving oxidative stress, mitochondrial dysfunction, innate immune activation, inflammasome signaling, and inflammatory cytokine production. These mechanisms reduce nitric oxide bioavailability, impair endothelial and smooth muscle function, promote fibrosis and tissue remodeling, and ultimately contribute to erectile dysfunction. Emerging evidence also implicates regulated cell death pathways, including pyroptosis and ferroptosis, as important mediators linking inflammation to cavernosal injury. Created in BioRender. Webb, C. (2026)

## Data Availability

No datasets were generated or analysed during the current study.
